# Physical trauma and injury: A multi-center study comparing local residents and refugees in Lebanon

**DOI:** 10.7189/jogh.11.17001

**Published:** 2021-10-09

**Authors:** Samar Al-Hajj, Mohamad A Chahrour, Ali A Nasrallah, Lara Hamed, Ian Pike

**Affiliations:** 1 Health Management and Policy Department, Faculty of Health Sciences, American University of Beirut, Beirut, Lebanon; 2 Department of Surgery, American University of Beirut Medical Center, Beirut, Lebanon; 3 Department of Pediatrics, Faculty of Medicine, University of British Columbia. BC Injury Research and Prevention Unit, BC Children’s Hospital Research Institute, Vancouver, BC, Canada

## Abstract

**Background:**

Refugees are susceptible to various types of injury mechanisms associated with their dire living conditions and settlements. This study aims to compare and characterize the emergency department admissions due to physical trauma and injuries among local residents and refugees in greater Beirut.

**Methods:**

This epidemiological study analyzes injury incidence and characteristics of patients presenting to Emergency Departments of 5 sentinel hospitals between 2017 and 2019. Using the WHO Injury Surveillance Guidelines and Pan-Asia Trauma Outcomes Study form, an injury data surveillance form was designed and used in hospital settings to collect data on injuries. Chi-square test analysis was performed to compare differences in injury characteristics between local residents and refugees. Regression models were constructed to assess the effect of being a refugee on the characteristics of injuries and outcomes of interest.

**Results:**

A total of 4847 injuries (3933 local residents and 914 refugees) were reported. 87.4% of the total injuries among refugees were sustained by the younger age groups 0-45 years compared to 68.8% among local residents. The most prevalent injury mechanism was fall (39.4%) for locals and road traffic injury (31.5%) for refugees. The most injured body part was extremities for both populations (78.2% and 80.1%). Injuries mostly occurred at home or its vicinity (garden or inside the camp) for both populations (29.3% and 23.1%). Refugees sustained a higher proportion of injuries at work (6%) compared to locals (1.3%). On multivariate analysis, refugee status was associated with higher odds of having an injury due to a stab/gunshot (odds ratio (OR) = 3.392, 95% confidence interval (CI) = 2.605-4.416), having a concussion injury (OR = 1.718, 95% CI = 1.151-2.565), and being injured at work (OR = 4.147, 95% CI = 2.74-6.278). Refugee status was associated with increased odds of leaving the hospital with injury-related disability (OR = 2.271, 95% CI = 1.891-2.728)]

**Conclusions:**

Injury remains a major public health problem among resident and refugee communities in Beirut, Lebanon. Refugees face several injury-related vulnerabilities, which adversely affect their treatment outcomes and long-term disabilities. The high prevalence of occupational and violence-related injuries among refugees necessitates the introduction of targeted occupational safety and financial security interventions, aiming at reducing injuries while enhancing social justice among residents.

Injury represents a leading cause of death and disability globally [1,2]. Annually, injury is responsible for over 5 million deaths, accounting for nearly 9% of global deaths [3]. Each year, millions of people sustain non-fatal injuries that require emergency department (ED) visits and hospitalizations, impacting individuals’ health and exerting financial pressure on health care systems [3]. The interplay of multiple intrinsic factors (eg, age, gender, education, socio-economic status) and extrinsic factors (eg, external environment, available and accessible health care service) strongly impact the frequency and severity of individual injuries [4-8]. Major discrepancies in the distribution of the global burden of injury-related mortality and morbidity are noted, with a substantial human and economic impact in low- and middle-income countries (LMICs) [9]. Due to the lack of injury preventive measures and sub-optimal health care services in these jurisdictions, over 90% of injury-related deaths occur in LMICs [10].

The Eastern Mediterranean Region (EMR) claims one of the highest global rates of unintentional fatal injuries among LMICs and the second leading cause of disability adjusted life years (DALYs) for youth aged 15-19 years [11,12]. Lebanon, an upper middle-income country in the Eastern Mediterranean region sustain a large toll of injury burden, as injury ranks 3^rd^ among leading causes of death and 5th in the leading causes of DALYs for the period 2000 to 2012 [13]. Additionally, regional wars and conflicts have exacerbated the injury profile in EMR countries including Lebanon, and presented additional factors that increase individual morbidity and mortality. The EMR, with its history of protracted political instability and regional wars, has witnessed the internal displacement of millions of families and individuals seeking refuge in neighboring countries. Lebanon endured frequent political unrest and conflicts, and reported high rates of injury morbidity and mortality [14,15] throughout its history. Recent regional war in neighboring Syria created an influx of refugees who crossed into Lebanon and settled in camps and informal settlements across the country, accounting for almost 1/3 of the population residing in Lebanon in 2015 - the highest number of refugees per capita in the world [16].

Refugee status represents a pivotal determinant in increasing individual exposure to injuries, particularly throughout their journey and settlements [17,18]. Review of existing literature demonstrates a discrepancy in the frequency and nature of injuries sustained by refugees compared to local residents [4,19-24]. In Lebanon, recent reports show that refugees sustain higher rates of injuries. For instance, in 2015, injuries accounted for almost 19.8% of hospitalization among Syrian refugees, compared to 14.9% among the local community [16]. Multiple interconnected factors aggravate refugee conditions and increase the risk of exposures to injuries, namely overcrowded living conditions, unsafe cooking, heating and lighting sources, lack of resources and poverty, and limited access to health care services. The literature is lacking a proper understanding of how refugee status is linked to increased vulnerability and affinity towards various types of injuries, particularly in the MENA region and Lebanon.

This study aims to understand the epidemiology and associated risk factors for injury among local and refugee communities in Beirut. It further aims to compare the rates of ED admissions due to injuries among local residents and refugees in greater Beirut, Lebanon. The generated evidence is essential to prioritize and adapt data driven injury prevention programs and policies.

## METHODS

### Study design

This study is a descriptive epidemiological analysis of injury cases that presented to the ED of 5 sentinel hospitals across the city of Beirut during the study period, June 2017 to May 2018. Sentinel hospitals expressed willingness to collaborate and provide timely injury data, and each hospital was selected based on its location in high population catchment areas. Participating hospitals were a mix of private and publicly funded, and each was in a distinct geographic location relative to other hospitals. The study population included all people who sustained an injury, whether intentional or unintentional, including poisoning, presenting at the participating study hospitals. Within the context of this study, local residents refer to patients with a Lebanese nationality while refugees refer to patients with a Syrian nationality.

### Data collection

Data were collected for individuals presenting with an injury at any of the participating hospitals within the 12-month period, from June 2017 to May 2018). The Pan-Asia Trauma Outcomes Study (PATOS) guided the design and development of a one-page injury data collection form for use in the ED at each of the hospitals to capture and quantify the characteristics of presenting injuries in this study [25]. The one-page PATOS data collection form was pilot tested at the primary site (American University of Beirut Medical Center) to ensure its feasibility prior to the full-scale data collection at the 5 participating sites. Data collectors were trained on the data collection form, and trained on ethics in Human Subjects Research (HSR) via the Collaborative Institution Training Initiative (CITI). At each hospital, patients’ ED medical records were filtered, and sampled based on the hospital monthly injury prevalence (number of injured cases/total ED visits) with a design precision of 5%-10% and a 95% confidence interval. Data were retrospectively reviewed, abstracted from patient charts by the trained data collectors, and entered electronically into a secure-password database that is accessed and managed only by authorized study investigators using RedCap electronic data capture tools [26]. All injury cases meeting the study criteria were de-identified, included and assigned a unique identifier, precluding the need for gathering any personal information. Injuries resulting in ED treatment and release, as well as those resulting in death (at ED arrival or shortly after presenting to ED) were all captured from ED patient records. Information collected included patients’ nationality, socio-demographic characteristics, injury epidemiology (intent, mechanism, location, nature, place, activity at time of injury), risk factors (alcohol, substance, seatbelt use), pre-hospital, ED, and hospital care, and injury outcome (death, hospitalization, treatment and discharge from ED).

### Statistical analysis

Statistical analyses were performed using the IBM SPSS statistical package (version 26, IBM Corp, Armonk NY, USA). Continuous data were reported as means and standard deviations, and comparisons were made using the independent *t* test. Categorical data were reported as counts and proportions with comparisons made using the chi-square test, or the Fisher exact test, as appropriate. Multivariable logistic regression models were constructed to assess the effect of refugee status on the characteristics of injuries and outcomes of interest, while adjusting for age, gender, intent and mechanism of injury. The results were presented as odds ratio (OR) and 95% confidence interval (CI). *P* < 0.05 was used to indicate statistical significance.

## RESULTS

A total of 4847 injuries were reported at the 5 participating hospitals during the period June 2017 to May 2018. More males (63%) than females sustained injuries that required an ED visit. This difference was greater among refugee males (68.7%) and females. The average age among Lebanese and Syrian patients was 35.1 (±23.9) and 27.0 (±17.7), respectively. Nearly eighty-eight percent (87.4%) of all injuries among refugees were sustained by the younger age groups (0-45 years) compared to 68.8% among Lebanese residents (*P* < 0.05), while 31.2% of injuries reported among Lebanese and significantly fewer (12.6%) among Syrian refugees were those aged 45+ years (*P* < 0.05). **Table 1** presents baseline characteristics of the patient population included.

**Table 1:** Baseline characteristics of patients presenting to the emergency department with injury, stratified by refugee status

Variable		Number (%)			*P-*value
**Overall**	**Lebanese**	**Refugees**
**Patients**		**4847**	**3933 (81.1)**	**914 (18.9)**
**Age**	Mean (SD)	33.6 (23.1)	35.1 (23.9)	27.0 (17.7)	**<0.001**
	Median (IQR)	28 (17-49)	29 (17-52)	25 (15-35)	**<0.001**
	0-<1	70	46 (1.2)	24 (2.6)	**<0.001**
	1-<15	999	809 (20.6)	190 (20.8)	
	15-<25	1007	772 (19.6)	235 (25.7)	
	25-<45	1429	1079 (27.4)	350 (38.3)	
	45-60	734	658 (16.7)	76 (8.3)	
	>60	608	569 (14.5)	39 (4.3)	
**Gender**	Female	1789	1503 (38.2)	286 (31.3)	**<0.001**
	Male	3058	2429 (61.8)	629 (68.7)	
**Marital status**	Married	909	829 (21.1)	80 (8.8)	**<0.001**
	Single	2363	1969 (50.1)	394 (43.1)	
	Unknown	1575	1135 (28.8)	440 (48.1)	
**Smoking status**	Smoker	756	654 (16.6)	102 (11.2)	**0.004**
	Non-smoker	2219	2003 (50.9)	216 (23.6)	
	Unknown	1872	1276 (32.4)	596 (65.2)	
**Insurance status**	Self	672	518 (13.2)	154 (16.8)	**<0.001**
	Private	2769	2475 (63.0)	289 (31.6)	
	Public/NGO	1406	935 (23.8)	471 (51.5)	
**Hospital type**	Public	4028	3554 (90.4)	474 (51.9)	**<0.001**
	Private	819	379 (9.6)	440 (48.1)	

SD – standard deviation, IQR – interquartile range, NGO – non-governmental organization

A significant difference was found in the hospital accessed for injury treatment and the insurance status between the two populations. Almost 90% of injured Lebanese sought treatment at private hospitals compared to almost half that proportion (51.9%) among refugees. The proportion of refugees treated at public hospitals (48.1%) was almost 5-fold higher than local residents (9.6%). This was similar for insured individuals, where 63% of the Lebanese population had private insurance compared with only 31.5% of Syrian refugees, who were mostly covered by funds made available through the United Nations High Commissioner for Refugees (UNHCR) and local and international NGOs (51.5%).

A significant difference was present in the mechanism of injury among local and refugee communities. The most common injury among Lebanese residents was fall-related injury (39.4%); higher than those reported among Syrian refugees (27.5%). Road traffic injuries (RTIs) were similar in both populations - 30.1% (Lebanese) and 31.5% (Syrian refugees). The majority of the injuries sustained by both communities were unintentional. The prevalence of assault (interpersonal violence) among refugees was 2.1%, almost double that sustained by residents (1.1%). Stab or gunshot injuries were significantly higher in the refugee population compared to locals (12.6% and 3.7%, respectively). Both populations experienced high frequencies of upper and lower extremity injuries (78.2% and 80.1%) followed by head and facial injuries (24.5% and 23.2%). Local residents and refugees experienced significantly different rates of injuries to the abdomen (1.9% and 3.6%, respectively).

The most common injury types sustained by locals were bruises/superficial injury and sprains/strains with 26.5% and 26.3% respectively compared to 20% and 19.5% among refugees. Similarly, fractures constituted a larger proportion of injuries among locals (24.1%) than among refugees (19.6%). Refugees had a significantly higher proportion of cuts/bites/open wounds and organ system injuries with 36.5% and 4.9% respectively compared to 22.1% and 2.6% among locals.

Home/garden/building (inside camps in the case of refugees) were the most common site of injury in both communities yet with slightly different proportions for locals (29.3%) compared to refugees (23.1%). Locals’ injuries occurred more often during leisure and sports-related activities (51.7% and 6.9% respectively), compared with refugees (33.6% and 3.2% respectively). Refugees, however, had a noticeably higher proportion of occupational injuries (12.4%) occurring at work sites; almost 2.5 times more than locals (4.9%). **Table 2** presents the difference in injury characteristics for the two populations.

**Table 2:** Injury characteristics, stratified by refugee status

Variable		Number (%)			*P-*value
**Overall**	**Lebanese**	**Refugees**
**Intent of injury**	Unintentional	4635	3783 (96.2)	852 (93.2)	**<0.001**
	Assault	61	42 (1.1)	19 (2.1)	**0.014**
	Intentional self harm	66	56 (1.4)	10 (1.1)	0.438
	Others	85	52 (1.3)	33 (3.6)	
**Mechanism of injury**	Fall	1797	1546 (39.3)	251 (27.5)	**<0.001**
	Fire, flame or heat	114	100 (2.5)	14 (1.5)	0.069
	Stab or gun shot	262	147 (3.7)	115 (12.6)	**<0.001**
	Road traffic injury	1470	1182 (30.1)	288 (31.5)	0.388
	Physical overexertion	373	319 (8.1)	54 (5.9)	**0.024**
	Others	357	264 (6.7)	93 (10.2)	<0.001
	Unknown	474	375 (9.5%)	99 (10.8)	
**Body part injured**	Head	574	468 (11.9)	106 (11.6)	0.799
	Face	601	495 (12.6)	106 (11.6)	0.414
	Neck	102	90 (2.3)	12 (1.3)	0.064
	Thorax	201	169 (4.3)	32 (3.5)	0.277
	Abdomen	109	76 (1.9)	33 (3.6)	**0.002**
	Spine	183	145 (3.7)	38 (4.2)	0.501
	Upper extremity	1889	1520 (38.6)	369 (40.4)	0.336
	Lower extremity	1921	1558 (39.6)	363 (39.7)	0.955
	Skin	45	37 (0.9)	8 (0.9)	0.852
	Other non-anatomical	25	12 (0.3)	13 (1.4)	**<0.001**
**Injury type**	Fracture	1126	947 (24.1)	179 (19.6)	**0.004**
	Strain/sprain	1211	1033 (26.3)	178 (19.5)	**<0.001**
	Cuts/bites/open wound	1205	871 (22.1)	334 (36.5)	**<0.001**
	Bruise	1225	1042 (26.5)	183 (20)	**<0.001**
	Burn	122	105 (2.7)	17 (1.9)	0.159
	Concussion	156	119 (3)	37 (4)	0.115
	Organ system	147	102 (2.6)	45 (4.9)	**<0.001**
	Other	11	7 (0.2)	4 (0.4)	0.137
**Location**	Home/garden/building	1362	1151 (29.3)	211 (23.1)	**<0.001**
	School/sports area	327	297 (7.6)	30 (3.3)	**<0.001**
	Street	537	433 (11)	104 (11.4)	0.749
	Industrial/construction/work	107	52 (1.3)	55 (6)	**<0.001**
	Others	193	167 (4.2)	26 (2.8)	0.051
**Activity**	Work	305	192 (4.9)	113 (12.4)	**<0.001**
	Education/school	15	14 (0.4)	1 (0.1)	0.227
	Sports	299	270 (6.9)	29 (3.2)	**<0.001**
	Leisure	2340	2033 (51.7)	307 (33.6)	**<0.001**
	Others	358	272 (6.9)	86 (9.4)	0.009

A significantly higher proportion of locals (10.3%) were admitted to hospitals compared with refugees (7.1%), while a larger proportion of refugees (5.6%) left Against Medical Advice (AMA) compared with locals (2.8%). While the majority of both locals (79.9%) and refugees (74.8%) left the hospital with no disability, the proportion of refugees (25.1%) leaving with moderate/severe disability was higher compared to the local population (19.9%). **Table 3** presents the differences in outcomes for the two populations.

**Table 3:** Disposition outcomes of patients presenting with injury, stratified by refugee status

Variable		Number (%)			*P-*value
**Overall**	**Lebanese**	**Refugees**
**Disposition**	Treated and discharge	4022	3311 (84.2)	711 (77.8)	0.562
	Admitted to hospital	469	404 (10.3)	65 (7.1)	**0.018**
	Transferred to another hospital	29	23 (0.6)	6 (0.7)	0.684
	Left AMA	161	110 (2.8)	51 (5.6)	**<0.001**
	Dead	2	1 (0)	1 (0.1)	0.301
**GOS at Discharge**	Recovering	3550	2951 (79.9)	599 (74.8)	**<0.001**
	Moderate/severe disability	935	734 (19.9)	201 (25.1)	**0.014**
	Vegetative/dead	8	7 (0.2)	1 (0.1)	0.941

AMA – against medical advice, GOS – Glasgow Outcome Score

**Figure 1:**
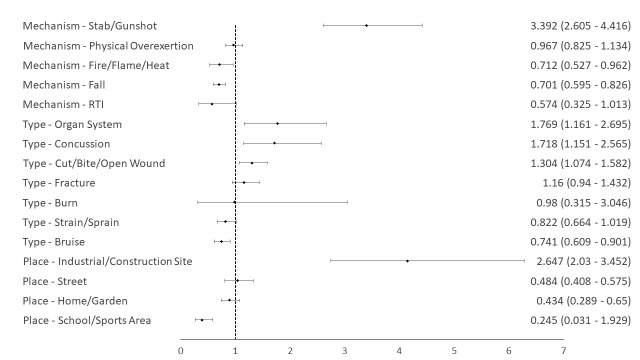
Forest plot showing adjusted odds ratios and 95% confidence intervals for Syrian refugee status as a risk/protective factor for each mechanism, type, and place of injury.

Multivariable logistic regression adjusting for age, gender, and intent of injury showed that refugee status was an independent risk factor for sustaining gunshot or stab injuries (odds ratio (OR) = 3.392, 95% CI = 2.605-4.416, *P* < 0.001), and a protective factor for sustaining a fall injury (OR = 0.701, 95% CI = 0.595-0.826, *P* < 0.001). When adjusting for age, gender, intent of injury, and mechanism of injury, refugee status was significantly associated with higher likelihood of sustaining cuts/bites/open wounds (OR = 1.304, 95% CI = 1.074-1.582, *P* = 0.007), concussion (OR = 1.718, 95% CI = 1.151-2.565, *P* = 0.008) and organ system injury (OR = 1.769, 95% CI = 1.161-2.695, *P* = 0.008) as well as lower odds for presenting with a bruise (OR = 0.741, 95% CI = 0.609-0.901, *P* = 0.003). Refugee status was associated with higher odds of injuries sustained at industrial/construction/work site OR = 4.147, 95% CI = 2.74-6.278), *P* < 0.001) and lower odds of being injured at school or sports areas OR = 0.393, 95% CI = 0.265-0.584, *P* < 0.001). As for outcomes, refugee status was associated with an increased likelihood of leaving the hospital with some form of disability (OR = 2.271, 95% CI = 1.891-2.728, *P* < 0.001). **Figure 1** shows a summary of the multivariable logistic regression analysis.

## DISCUSSION

This study compares injury characteristics, clinical disposition and risk factors among Lebanese and refugee communities in the capital city of Beirut, Lebanon. Evidence from this study reveals distinct and relatively heterogeneous patterns of injuries and outcomes between residents and refugees. This disparity provides insights into understanding the injury exposure and types of injuries sustained by both communities and allows for the design of tailored injury prevention and safety programs.

Consistent with existing studies, a predominance of male injuries in both resident and refugee communities was noted, with a slightly larger proportion of injuries among male refugees (68.7% vs 61.8%) [7,27]. The refugees’ male workforce exposure to hazardous occupations in industrial or construction sites may increase their vulnerability to injuries. Refugees had more than 4 times higher odds of presenting at an emergency department with an injury sustained at the workplace compared with locals. Refugee’s harsh working conditions and lack of proper training at workplaces have been reported as major contributing factors to this notable disparity [28]. In particular, refugees mostly work as construction workers in Lebanon, with hazardous work environments and an evident lack of proper safety measures adopted at construction and industrial sites [29-32]. Similar evidence has been noted in a recent Canadian study indicating that refugees and recent immigrants have a higher risk of occupational safety hazards as they are less likely to receive information on safety and health or undergo formal job training [7]. Refugees typically performed more physically demanding jobs without the use of proper safety protection gears, which increase their risk for injury [24]. Similarly, in Germany, Spain, and France, the incidence of work-related injuries is higher among migrants compared to citizens, particularly in jobs in industrial, construction, and agricultural sectors [33]. Refugees’ higher odds of presenting with concussion or organ system injury noted in this study may be traced to the severe injuries occurring at construction sites, and result from such things as falling from a height. Higher prevalence of traumatic brain injury (TBI) has been previously documented in refugee populations [18] in Denmark and the United Kingdom, leading to severe outcomes and long term disabilities [34,35].

A considerable variation in injury cases by age group and activities existed among residents and refugees. While residents experienced a more diverse pattern of injury, distributed evenly across all age groups, refugees sustained a high burden of injuries in age groups that might be considered productive, with a limited number of injuries reported among the geriatric population. Refugees younger than 25 years of age experienced the larger proportion of injuries, in line with previous studies indicating that the rate of unintentional injuries among children and youth was 20% higher in refugees compared to non-refugees [7]. This trend may be explained by refugees’ poor living conditions, overcrowded housing and inappropriate child care [27].

A significant difference between the refugees and local community was present in the access to care at public vs private hospitals. Compared to public hospitals, Lebanese private hospitals typically maintain adequate resources and infrastructure, reflecting enhanced patient services and provision of care [36]. Only 9% of locals in the cohort sought care at public hospitals compared to almost 50% of the refugee population. As refugees more often lack the means for out-of-pocket expenses to cover medical services, they are forced to seek health care services at public hospitals and selected health care facilities subsidized by UNHCR and local NGOs [37]. This highlights the issue of limited availability and accessibility of health care services among refugees. With the limited public health care system in Lebanon, refugees’ increased rates of non-communicable diseases and larger burden of injury places substantial strain on local system resources and often exhausts its capacities [38,39].

Fall and road traffic injury were among the leading causes of injuries in both populations with a slight variation in age group distribution. Lebanese locals were at a higher risk of sustaining fall-related injuries, demonstrated by the adjusted odds ratio of 0.7 that confirmed the decreased risk of fall-related injuries among refugees. Further analysis indicates that over 72% of the elderly Lebanese population suffered from fall-related injuries, consistent with current literature which underscores the propensity of older adults to suffer from fall injuries [40-42]. Road traffic injury represents another major contributor to injuries in both resident and refugee communities. While this aligns with regional data showing a similar high proportion of emergency visits due to road traffic injuries, it is divergent to studies conducted in China and Canada showing increased risk for suffering from motor-vehicle accidents and severe traffic injuries in refugee communities compared to non-refugees, as well as to a study conducted in Turkey showing an opposite higher proportion of road traffic injuries among locals [7,23,27]. Road traffic injuries, hence, highly depend on the specificities of the local environment and its built-in safety infrastructure. In Lebanon, the absence of road safety measures coupled with the lack of compliance and enforcement, represent major contributing factors responsible for the high rates of road traffic injuries sustained by both local and refugee communities alike [43].

Even though most injuries were unintentional in both populations, assault injuries reported by refugees constituted a 2-fold higher proportion than that sustained by locals. Nonetheless, these numbers could have been subject to a reporting bias as patients presenting to the emergency department may hide the true intent of injury to avoid possible police investigations, especially as many refugees seek refuge in Lebanon unlawfully. This could be further delineated by the high proportion of stabbing and gunshot injuries among refugees constituting 12.6% of all injuries compared to 3.7% among the local population. The increased risk of assault injuries among refugee communities has been previously documented [21], and may be explained by multiple factors including poverty and possible criminal gang involvement [44,45]. Refugees, having been subject to trauma, instability and displacement stressors, are exposed to accumulated mental disturbances, leading to an increased tendency toward assault and violence [46-48].

Patient dispositions and outcomes varied considerably between the two populations. Refugees had almost 2.3 times greater odds of being discharged with a Glasgow Outcome Score (GOS) of moderate to severe disability compared to locals. The increased injury morbidity and mortality among the refugee community is often shaped by a combination of multiple factors, including unsafe living conditions, hazardous working environments, and limited access to health care services [49]. While the worse outcomes could be due to initially more severe injuries, this high proportion raises a major concern regarding the environment in which refugees live and work, and the quality of health care service they access. The potential discrepancy in severity of injury on presentation could be due to a higher exposure of risky settings and violence, as well as the lack of access to immediate health care services which exacerbates the injury severity and affects outcome due to delayed and sub-optimal health care services [50]. In the same context, almost double the proportion of refugees elected to leave the hospital Against Medical Advice (AMA) compared to locals, further elucidating refugees’ limited access to health care and possible inability to afford hospital admission associated health expenditures [49].

A series of recommendations aiming at decreasing injuries among the refugee community can be presented based on evidence provided from this study. First and foremost, protective policies and procedures should be implemented to prevent workplace injuries and to safeguard workers’ occupational health and safety [24]. Second, appropriate work training and injury awareness programs should be integrated to increase safety at the workplace. Third, concerted efforts should focus on designing safe and appropriately populated refugee camps, in addition to developing and delivering awareness and educational activities at camp sites that aim to educate refugees and raise awareness about common injuries sustained at camps. Fourth, to ease the problem of violence and assault within the refugee community, efforts should be focused on mitigating poverty and alleviating mental health problems. Securing job opportunities and providing refugees with financial support can safeguard them from seeking illegal channels of money, and render them less susceptible to be involved in violence and assault [51,52]. Similarly, working on refugees’ mental well-being can help to alleviate the psychiatric conditions that might perpetuate the tendency to engage in violent acts within refugee communities [53]. Finally, more resources should be secured to ensure refugees’ easy access to the health care services in the country.

To the best of our knowledge, this study is the first that compares the various factors, characteristics, and outcomes of injuries sustained by refugees and locals residing in Lebanon. The study, however, is not without limitations. First, the study is a retrospective and may be hindered by the availability of certain injury-related variables. Second, a methodological limitation might be introduced with the inclusion of all patients with Syrian nationality under the group of refugees, as a minor proportion of those could have been living in Lebanon even prior to the onset of the Syrian war. Third, the absence of the injury severity score at ED presentation limited the ability to accurately assess patients’ treatment outcomes. Moreover, as socioeconomic status may play a role in injury characteristics and outcomes, having access to household income data may have proved helpful in further stratifying the analysis, and is encouraged in future studies of this nature. Nonetheless, with data pooled from 5 public and private hospitals covering all age groups, this study captured a representative sample of the local citizen and refugee populations, increasing our understanding of the types and risks of injury, and enhancing its generalizability in recommending injury prevention strategies.

## CONCLUSION

Injury remains a major public health problem among resident and refugee communities in Beirut, Lebanon. Refugees face several injury-related vulnerabilities due to their harsh living and working conditions coupled with their limited access to health care services, which adversely affects their treatment outcomes and long-term disabilities. The high prevalence of occupational and violence-related injuries among refugees necessitates the introduction of targeted occupational safety interventions, aiming at reducing injuries while enhancing social justice among residents.
